# Internet of Things Concept in the Context of the COVID-19 Pandemic: A Multi-Sensor Application Design

**DOI:** 10.3390/s22020503

**Published:** 2022-01-10

**Authors:** Alexandru Lavric, Adrian I. Petrariu, Partemie-Marian Mutescu, Eugen Coca, Valentin Popa

**Affiliations:** 1 Computers, Electronics and Automation Department, Stefan Cel Mare University of Suceava, 720229 Suceava, Romania; apetrariu@usm.ro (A.I.P.); marian.mutescu@usm.ro (P.-M.M.); eugen.coca@usv.ro (E.C.); valentin.popa@usm.ro (V.P.); 2 MANSiD Research Center, Stefan Cel Mare University of Suceava, 720229 Suceava, Romania

**Keywords:** Internet of Things, COVID-19 sensors, remote healthcare, telemedicine, quarantine monitoring, IoT wearable device

## Abstract

In this paper, we present the design, development and implementation of an integrated system for the management of COVID-19 patient, using the LoRaWAN communication infrastructure. Our system offers certain advantages when compared to other similar solutions, allowing remote symptom and health monitoring that can be applied to isolated or quarantined people, without any external interaction with the patient. The IoT wearable device can monitor parameters of health condition like pulse, blood oxygen saturation, and body temperature, as well as the current location. To test the performance of the proposed system, two persons under quarantine were monitored, for a complete 14-day standard quarantine time interval. Based on the data transmitted to the monitoring center, the medical staff decided, after several days of monitoring, when the measured values were outside of the normal parameters, to do an RT-PCR test for one of the two persons, confirming the SARS-CoV2 virus infection. We have to emphasize the high degree of scalability of the proposed solution that can oversee a large number of patients at the same time, thanks to the LoRaWAN communication protocol used. This solution can be successfully implemented by local authorities to increase monitoring capabilities, also saving lives.

## 1. Introduction

In recent years, we have witnessed continuous discussions about the IoT (Internet of Things) concept, which involves the connection of various objects that surround us in our everyday life to the Internet. The main purpose of the IoT concept is closely related to the smart city topic, enabling an increase in quality of life by contributing to the efficient use of resources and environment protection.

IoT technologies are sufficiently enhanced to enable the development of integrated solutions for the challenges humankind is facing today. All this knowledge and technological progress does not seem to have prepared us enough for the current context of the pandemic we are experiencing. Considering the pandemic context, all of these IoT devices that surround us in our daily life are powerless in the face of the spread of the SARS-CoV2 virus infection. Until the start of the pandemic, there was not a strong connection between the IoT and the healthcare industry. This aspect must change and allow for the integration of healthcare services in current existing IoT infrastructure; this change is also forced by the current pandemic situation. Thus, it is our responsibility, as a research community, to develop new systems and find solutions to current problems.

The COVID-19 disease, which is a severe acute respiratory syndrome generated by the SARS-CoV2 virus, is an ongoing pandemic [1]. This has led to an immense public health concern in the international community, as the World Health Organization (WHO) has stated, that the outbreak was a public health emergency. International concern led to declaring the COVID-19 outbreak a global pandemic on the 11th of March 2020 [2]. Since then, things have progressed quite rapidly, with the number of infections growing exponentially. Globally, as of 8th December 2021, there have been approximately 300 million confirmed cases of COVID-19, including more than 5 million deaths, reported to WHO [3].

In this paper, we present the design, development, and implementation of an integrated system for COVID-19 illness management that is associated with the SARS-CoV2 virus infection. The urgent need for this system is related to the fact that the architecture can enable local authorities to reduce the pressure on the medical system by integrating telemedicine facilities allowing for remote monitoring of patients. Thus, the health status of patients is closely monitored by medical staff and depending on the current load on the medical system, suspect patients, quarantine, isolated patients and even mild cases can be remotely monitored while at home. 

The proposed system integrates an IoT multi-sensor approach that offers the possibility of monitoring different vital signs of the patient. The patient-monitoring node is integrated in a wearable device that is easy to wear and fully reconfigurable, allowing further development by integrating other sensor types. Thus, the proposed system can be seen as a powerful IoT multi-sensor platform. The implemented and tested novel architecture allows for the integration of a high number of wearable devices, in an effective and cost-efficient manner. The system also monitors the persons that are quarantined due to infection or suspicion of infection with the SARS-CoV2 virus.

The paper is organized as follows: first, a brief introduction related to the state-of-the-art technology, followed by Section 2, where the main IoT challenges regarding the SARS-CoV2 pandemic are presented. The IoT communication protocols, and challenges for pandemic control are discussed in Section 3. In Section 4, the IoT wearable multi-sensor architecture for remote patient monitoring is presented and discussed in detail. The final section of the paper is represented by the conclusions and the overall performance discussion of the system. From the analysis conducted in this paper and the obtained results, we can see that the proposed system ensures a high-level of performance and can be easily developed and implemented by the local authorities to scale up the remote patient monitoring capabilities, ultimately saving lives.

The main contributions and originality of this paper are the following:

(1) The design of an in-depth analysis of IoT challenges regarding the SARS-CoV2 pandemic;
(2) The performance evaluation of the communications protocol that can be integrated in the patient monitoring architecture distributed over a large geographical area;
(3) The design, development, and implementation of a novel integrated system for COVID-19 illness management;
(4) The patient monitoring IoT multi-sensor platform integration in a wearable device that is easy to wear and fully reconfigurable;
(5) The multi-sensor IoT wearable device can monitor the health condition related parameters like pulse, blood oxygen saturation and body temperature, as well as the patient’s current location increasing the monitoring capabilities of the local authorities and saving lives.

## 2. SARS-CoV2 and IoT Integration

The SARS-CoV2 virus infection can be the cause of a potentially fatal disease that currently threatens humanity as a global public health issue. The rapid person-to-person transmission of COVID-19 infection is extremely concerning. It is precisely this aspect that led to many people being isolated or quarantined. Thus, extensive measures to reduce person-to-person transmission have been implemented to control the current outbreak. Special attention and significant efforts to protect or reduce transmission must be applied in sensitive populations, including children, healthcare providers, older people, and people with serious health issues. 

The new B.1.1.529 or Omicron variations of SARS-CoV2 may be more transmissible than other variants and are partially resistant to existing vaccines. According to Markets and Markets, the Internet of Medical Things (IoMT) market is currently worth USD 26.5 billion and is expected to reach USD 94.2 billion by 2026 with a CAGR (Compound Annual Growth Rate) of 28.9% [4]. The major challenges in using the IoT concept are most often related to ensuring a high autonomy (obtaining the lowest possible energy consumption), the possibility of integrating as many wireless sensors as possible, scalability, and ensuring the highest possible communication range. 

Another aspect that should not be neglected is that of communication system deployment costs, that should be kept to a minimum. Thus, it would be ideal for the sensors to be able to transmit data within an unlicensed frequency spectrum, avoiding additional costs required by cellular data subscriptions. Nowadays, IoT technologies are mature enough to be integrated into healthcare diagnostic systems that can contribute effectively to the fight against this unseen enemy named COVID-19. All these aspects have generated a series of new problems that we have never faced before. Thus, it is our duty to identify new solutions that contribute to increasing the patient’s medical care by providing support to local government authorities.

**Figure 1:**
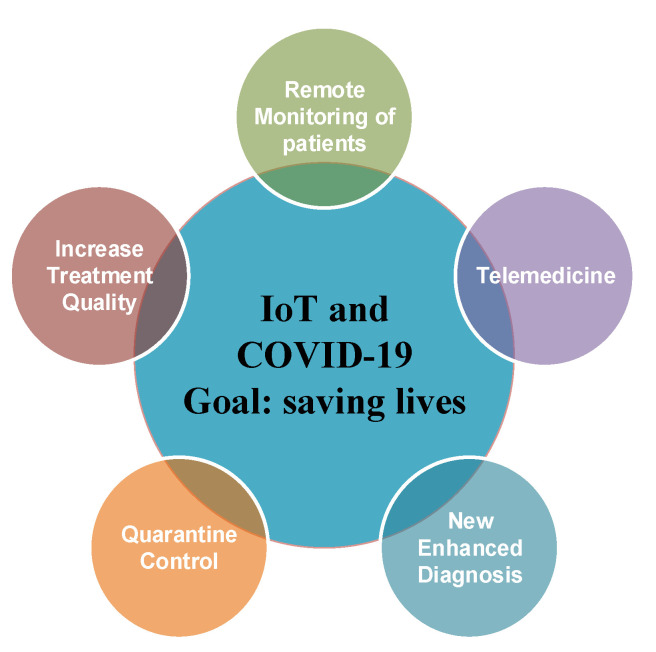
Advantages of using IoT for COVID-19 pandemic monitoring.

Worldwide, there are several applications that use machine learning (ML) technologies into real-time healthcare decisions, contributing a great deal to saving lives. Improving the efficiency and quality of hospital care services has proved to be an important and critical challenge during the pandemic. Machine learning is also included in the informational technology family along with artificial intelligence (AI) [5,6,7,8], as well as AI prediction [9] and it can be integrated within the diagnostic process, contributing to the rapid detection of the SARS-CoV2 infection [10,11]. These mechanisms can also help reduce misdiagnosis, reduce the diagnosis time and ultimately save patients’ lives. All these techniques along with the above-mentioned mechanisms must help in the context of the pandemic and contribute to slow down the spread of infection and make the diagnosis process more efficient. Figure 1 presents the main identified advantages of using IoT devices to fight against the current COVID-19 pandemic. The advantages that can contribute to saving lives are related to the increase in treatment quality [12], remote monitoring of the patients [13], quarantine control systems [14], telemedicine [15], new enhanced diagnosis techniques and improving the virus detection techniques [16].

During the pandemic, local authorities also faced situations where people violate the imposed quarantine or isolation conditions. Thus, the designed system proposed in this paper allows for the active monitoring of the patient’s position while also monitoring the health condition. The main advantages are to limit the spread of the disease and avoid serious complications by administering the optimal treatment on time.

## 3. Communication Protocols, and IoT Challenges for Pandemic Control

The current situation demands a deep analysis for a better understanding of humankind’s preparation level in facing the SARS-CoV2 virus. This analysis was performed taking into account the informational technologies that can be used and integrated into applications and systems which will allow the flattening of the pandemic evolution curve and can even help eradicate it. Humankind has invested enormously in a variety of technologies that surround us in our daily life, contributing to the increase in its quality, but it seems that in the face of such an enemy we are powerless. 

The introduction of communication capabilities to simple objects involves the development and implementation of large-scale, high-density complex network topologies. Usually, the wireless sensor devices are distributed over a large geographical area so specialized communication protocols are needed to integrate a large number of devices in the same network. All these protocols must also ensure a high level of performance and be cost efficient. 

The first challenge when a large-scale patient monitoring system is developed is related to the selection of the communication protocol. Thus, to select the best candidate for the communication protocol, functional requirements of the system must be correlated with the current available technologies. The second step is related to the design and development of a wearable IoT multi-sensor platform that can integrate a multi-sensor approach in a flexible manner, while also considering the power efficiency of the design.

Currently, there are all sorts of standards, protocols, and communication mechanisms that promise to solve the main problems raised by the IoT concept like SigFox [17], NB-IoT [18], Symphony Link [19], IEEE 802.15.4 [20], Z-Wave [21], IEEE 802.15.1 [22] and cellular technologies [23]. Table 1 contains a comparison of the main IoT communication protocols that can be implemented in the IoT patient monitoring system.

The particularity of the patient monitoring system involves the integration of many sensors that can communicate and are distributed over a large geographical area. The challenges are great considering the small communication distance due to the limited access resources of sensors like processing capabilities, available data storage or limited power sources. At the same time, to ensure the highest possible level of performance, as well as the integration of multiple sensors, it is mandatory that we improve and try to enhance the communication mechanism through intense research while constantly evaluating the possibilities it offers in stopping the current pandemic context.

To summarize, the communication protocol that can be integrated in the patient monitoring architecture has to meet the following characteristics:

➢ Ensure a long communication distance and a high-level of performance within a city-specific architecture;
➢ Have the possibility to integrate wireless sensors distributed over a large geographical area;
➢ Be suitable for non-LoS (line of sight) urban specific conditions;
➢ Have resistance to radio interference;
➢ Confirm the technology is already available and implemented by many municipalities;
➢ Facilitate massive deployment within a low complexity, communication architecture, with a short implementation time;
➢ Contribute to the active monitoring of the patient’s health condition;
➢ Have low-power consumption to allow the integration in a wearable multi-sensor smart device;
➢ Provide a high level of performance as a cost-effective solution.

From the previously presented information we can conclude that the LoRaWAN communication protocol ensures a high level of performance and can be integrated in development of the patient monitoring system. 

LoRaWAN is a communication protocol for media access control (MAC) designed for IoT applications and wide area networks that use LoRa modulation. LoRa modulation uses orthogonal spreading factors (SF) for individual wireless nodes to increase the communication range by reducing the data rate. LoRa modulation uses communication channels with fixed bandwidths of 125 kHz or 250 kHz for uplink channels and 500 kHz for downlink radio channels [24]. The SF can be varied from 7 to 12. The higher the SF, the higher the value of the packet time on air (ToA) will be, lowering the data rate and increasing the communication range by lowering the sensitivity level of the radio transceiver, from −123 dBm for using SF7 to −137 dBm when SF12 is used, respectively [25]. Thus, LoRa wireless nodes that are located in the proximity of the gateway will use SF7 and nodes located at longer distances will use SF12. The SF allocation is defined by the LoRaWAN specifications [26] in an adaptive data rate (ADR) algorithm where the SF is increased if the communication link budget is high meanwhile the SF is reduced if the communication link budget is low. The ADR and SF particularity of the LoRaWAN communication protocol offers a real advantage in urban environmental conditions, achieving long communication distances and is ideal for the patient monitoring system. 

The increase in COVID-19 infected people brought along with it a general overload of the medical system. In many regions the hospitals are overwhelmed with severe cases, leaving no room for the mild cases. Taking this into consideration, recently we have seen great interest in remote patient monitoring devices and in telemedicine, as is discussed in the previous section. In the scientific literature, multiple approaches are presented for remote patient monitoring, each one with unique features, advantages or disadvantages that must be overcome in future iterations.

Zhang et al. [27] present a device for COVID-19 prevention that monitors and records the daily activity of a patient. The main monitored parameters are the movement of the body recorded with a 3-axis accelerometer and the body temperature recorded with a CMOS analog temperature sensor. The device is attached to the subject’s wrist with two elastic bands to ensure constant contact of the temperature sensor. The data are transmitted to a PC using a BLE (Bluetooth low energy) module. The system also integrates an AI approach as to detect patterns of human activities. One disadvantage of the system is the short range of the communication transceiver, covering only approximately 10 m. The developed architecture is not scalable due to the BLE integration thus, long-distance communication was not considered.

Ullah et al. [28] propose a patient quarantine monitoring system based on multiple sensors approach. The sensors used in the application (temperature, respiratory, accelerometer, pulse, SpO_2_ and GPS—global positioning system) are distributed on the patient’s body. The communication with the main microcontroller unit (MCU) is achieved using a BLE communication link, same as the above related solution. The MCU forwards the measurement information to a local server through an Internet connection. The system provides a solution for the indoor GPS signal limitation by measuring the RSSI (receive signal strength indicator) of the Wi-Fi signal between the sensor device and the locally deployed server. Alerts and notifications are sent to the local authorities if the quarantine conditions are violated. This system is presented as being able to monitor multiple patients, but the capability is reduced due to the low coverage area of the Wi-Fi communication. Another disadvantage is the high complexity of the system on the sensor level, as the sensors are distributed on different areas of the body being mostly a proof-of-concept system, unavailable as an integrated wearable device.

Mukhtar et al. [29] present an IoT enabled solution for patient monitoring, using a rule-based approach for determining the current state of the patient health. As with the previous presented system [28], the sensors are distributed on the patient’s body to collect data, regarding the pulse, SpO_2_, temperature and cough rate. The measurements are sent to the cloud by an 802.11n communication protocol (Wi-Fi), where they are processed and analyzed. The authors define four patient classes, each patient being attributed a class through a rule-based classification process. The classes are summarized in Table 2.

A similar patient monitoring system is presented in [30]. The system uses an accelerometer sensor and two temperature sensors (a contact one and an IR—infrared sensor) for movement and body temperature measurement, respectively. Additionally, the system includes an ambient temperature and a humidity sensor to detect and warn the patient if the room parameters are off the limits. The measurements are sent to the web-based application via MQTT (message queue telemetry transport) using a BLE connection. The whole system is implemented into a M5stickC device, being an off-the-shelf embedded solution for a multi-application fast prototyping system.

Some published works propose COVID-19 tracing applications using Bluetooth low energy in order to track and monitor the spread of the virus [31,32]. These applications enhanced with ML capabilities can determine persons that are at risk of being infected because they were in the proximity of a confirmed infected person.

During our evaluation regarding the literature survey, three major disadvantages have been identified, one being related to the communication protocol used for patient monitoring systems. In this category, the lack of scalability due to the low communication range of the technology used to send the sensor data to the monitoring center, is a serious problem, which is not available when LoRaWAN protocol is used. Another disadvantage is related to the lack of energy efficiency of the proposed systems because they use a constant communication link, that drains the battery of the monitoring device very fast. The last major disadvantage is related to the hardware architecture modularity. Few monitoring systems use sensors in a wearable device that are comfortable and easy to wear. Thus, most of the solutions are related as a proof-of-concept design, being impossible to add new sensors to extend their functionality.

## 4. IoT Multi-Sensor System for Remote Patient Monitoring

**Figure 2:**
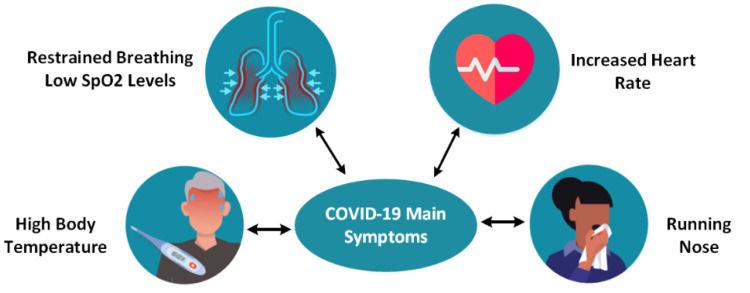
COVID-19 disease symptoms.

This section presents the design of an IoT multi-sensor patient monitoring system that uses and integrates information technologies related to the IoT domain. The functionalities of the system are also described in detail. Figure 2 presents the main COVID-19 symptoms [33,34]. To ensure a high level of performance, these main symptoms must be monitored remotely so that the lives of quarantined patients are not endangered. The sooner a patient receives treatment, the lower the risk of developing severe life-threatening complications.

**Figure 3:**
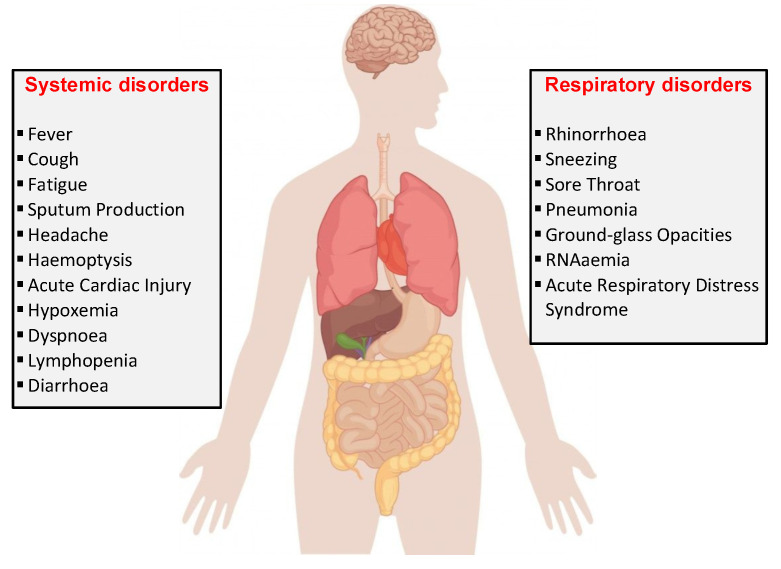
COVID-19 effects on the human body.

According to the CDC (Centers for Disease Control and Prevention), the main symptoms may appear on average 2–14 days after the exposure (based on the incubation period of MERS-CoV viruses), the symptoms being fever, cough [33], and shortness of breath due to impaired lung capacity [34]. Figure 3 shows some of the symptoms caused by the SARS-CoV2 virus. The latest studies show that the incubation period is about 5.2 days, so quarantine monitoring is crucial [35,36]. 

### 4.1. Patient Monitoring System 

Considering the previously identified communication protocol requirements, the LoRaWAN protocol [37,38] meets performance criteria such as high coverage radius, low costs, the possibility of integrating a large number of nodes, and developing a scalable system that can comply with the requirements of high-density wireless sensor network scenario suitable for urban non-LoS conditions.

**Figure 4:**
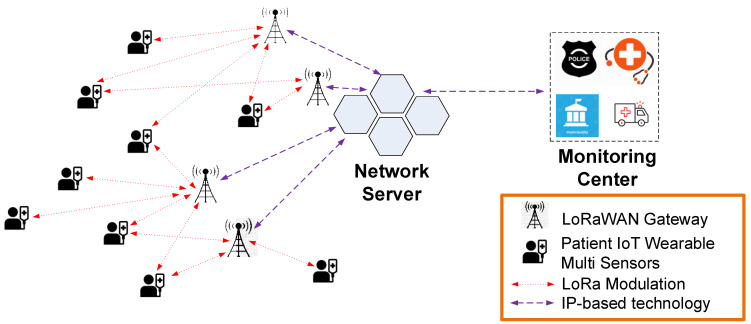
COVID-19 IoT multi-sensor patient monitoring architecture.

Figure 4 presents the proposed system architecture, which includes, the LoRaWAN gateway (GW), the sensors installed in the wearable device that monitors the patient’s vital signs and the network server (NS) that acts as a relay between the LoRaWAN network and the local monitoring center. All the collected data are stored at the monitoring center where medical staff oversee the health status of the patients. The information is conveyed through the LoRa modulation technology for long communication distances between the GW and the patient’s wearable device using IP-based technologies for the data transmission between multiple GWs and the NS.

The LoRaWAN architecture consists of LoRa transceivers installed in the IoT multi-sensor wearable devices and GW modules that are communicating directly with the NS. The integrated IoT multi-sensors send management and configuration commands to the LoRa transceiver whose main purpose is to transmit the message to the connected GW module. Since the communication protocol is an aloha-type wireless sensor network, end-devices are allowed to transmit arbitrarily [39]. The transceiver integrated in the IoT multi-sensor device is of class A type, meaning the communication is initiated only from the multi-sensor node side. This ensures that the energy consumption of the multi-sensor node is the lowest possible from all LoRaWAN communication classes available, allowing for long-term operation.

The main compromise of the LoRaWAN communication protocol is the limited time intervals in which the node can receive messages from the application server, but this mechanism is not an issue. Thus, after a message is sent to the NS, the node listens for any server messages only for a limited time interval. This disadvantage does not reduce the level of performance for the designed architecture.

Many local municipalities worldwide have already installed or have access to the city developed infrastructures so the wearable devices can be easily enrolled and added as an extension to the existing configuration with no supplementary costs. The medical staff can easily monitor many patients, and if a patient’s condition worsens, rapid intervention is possible. If the LoRaWAN communication protocol is used, then the system has no additional licensing costs or monthly fees like other communication protocols presented in Table 1, so the maintenance cost for the proposed system is low.

The system can be used both in institutionalized quarantine areas and in the case of people who are in solitary confinement at home. By setting up automatic alerts, a very large number of people can be monitored by a small number of medical staff in a centralized manner. Thus, we provide smart usage of the limited resources available in the pandemic context, contributing to saving as many lives as possible.

### 4.2. Wearable IoT Multi-Sensor Device for Patient Monitoring

**Figure 5:**
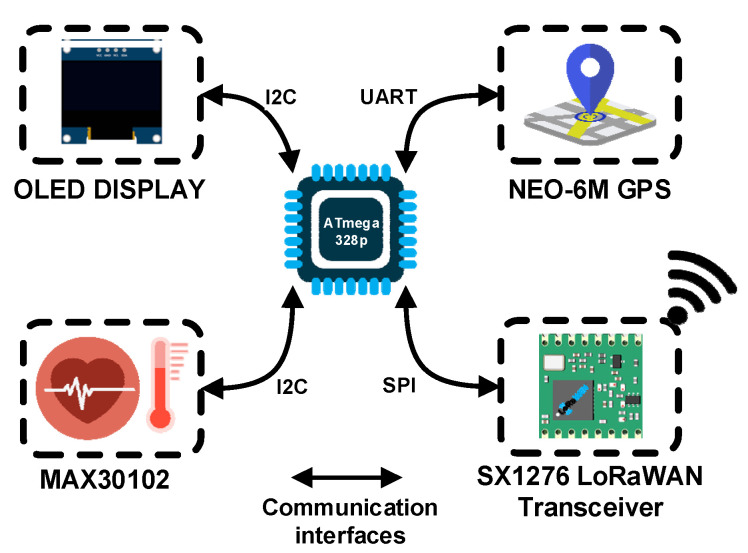
Wearable device block diagram of the multi-sensor approach.

Figure 5 presents the block diagram of the wearable device that includes an OLED display, the GPS sensor of Neo-6M type, the MAX30102 vital signs measurement sensor and the SX1276 LoRaWAN transceiver that allows for the collected data to be transmitted at the monitoring center. The wearable IoT device can monitor health conditions related parameters like pulse, blood oxygen saturation and body temperature as well as the patient’s current location. At the center of our wearable IoT multi-sensor device we have the ATmega328p microcontroller unit that processes the information collected from different sensors linked with I2C, SPI or UART busses.

For the health-related measurements, the MAX30102 [40] sensor was used as it offers an integrated solution for all three body parameters: pulse, blood oxygen saturation and body temperature, respectively. The sensor is based on the principle of spectrophotometry, which means that it detects the pulse and the blood oxygen saturation by measuring the amount of red and infrared light absorbed by the deoxygenated and oxygenated blood using photodiodes. The body temperature is measured using the temperature sensor integrated into the MAX30102. The signal provided by the MAX30102 is sent to the MCU via the I2C (Inter-Integrated Circuit) communication bus to be processed. The acquisition rate may be adjusted from the monitoring center based on the patient’s health status. Different patient classes can be implemented as presented in Table 2.

Monitoring the patient’s GPS position is also mandatory since maintaining a quarantine period involves isolation. Thus, a GPS module, named NEO-6M, is integrated into the sensor’s architecture, being connected to the MCU via the UART (universal asynchronous receiver-transmitter) communication interface. The location data are sampled at random time intervals as to obtain energy efficiency. By monitoring the patient’s current position, the authorities can be alerted in the eventuality that the patient does not comply with the quarantine conditions. If the current GPS position changes, the authorities are notified by a standard alert message transmitted to the monitoring center. 

**Figure 6:**
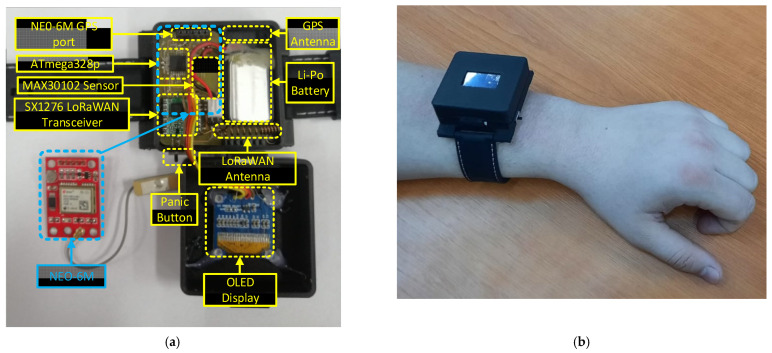
Physical implementation of the wearable device. (**a**) IoT multi sensor configuration. (**b**) IoT wearable device installed on patient.

The wireless transceiver used for long-range communication and LoRa modulation is SX1276 from Semtech [41]. The SF is adjustable using the ADR mechanism provided by LoRaWAN specifications, thus, a high-level of performance and power consumption optimization is obtained. Another feature of the wearable device is an OLED display used only for local parameter checks or a debug option in the testing scenarios. The monitoring platform is implemented in a compact wearable form factor case that can be attached to the patient’s wrist and is easily worn. Using a Li-Po power cell of 400 mA, the designed wearable device can operate for up to 20 days without recharging the battery, so the patient can wear the device during the 14-day quarantine period without power loss issues. This is possible by using a power management algorithm integrated into the MCU. The following rule can be used; for COVID-19 confirmed patients the time between measurements can be lower and offer more in-depth information about the evolution of the case. The physical implementation of the IoT multi-sensor wearable is presented in Figure 6.

Depending on the monitored case, by using the application from the monitoring center the local authorities can set a minimum predefined period per day for the wearable device to be worn. Thus, the proposed wearable multi-sensor device can be removed by the patient for short intervals. The developed system triggers alerts when the health of the person in quarantine deteriorates. Thus, the onset of symptoms should be closely monitored so that treatment is administered as soon as possible. The medical staff can adjust, from the monitoring application, different threshold values that can be applied individually per each monitored vital sign. These threshold values of monitored parameters allow the system to generate only relevant alarms and notifications, excluding false triggers. Additionally, the monitored person can make an emergency call for urgent medical services in case of rapid deterioration of their health status. This is achieved by simply pressing the panic button on the device worn by the patient. After the IoT wearable device joins the network, it starts monitoring the parameters: GPS location, heart rate, oxygen saturation level, and temperature of the patient. 

### 4.3. LoRaWAN Gateway Placement and Field Tests 

The LoRaWAN gateway module (GW) allows for the connection of a very large number of wireless sensors. This is one of the main advantages that determined the selection of LoRa technology as being at the center of the proposed system and used as the communication protocol. The optimal placement of the GW module is very important and has an impact on the global performance of the patient monitoring system. In order to determine the optimal position of the GW, RadioPlanner [42] software was used with the propagation models presented in [43]. The developed model included the urban geographical area that we wanted to monitor, the terrain configuration, building positions, heights, and other interferences including vegetation path losses.

**Figure 7:**
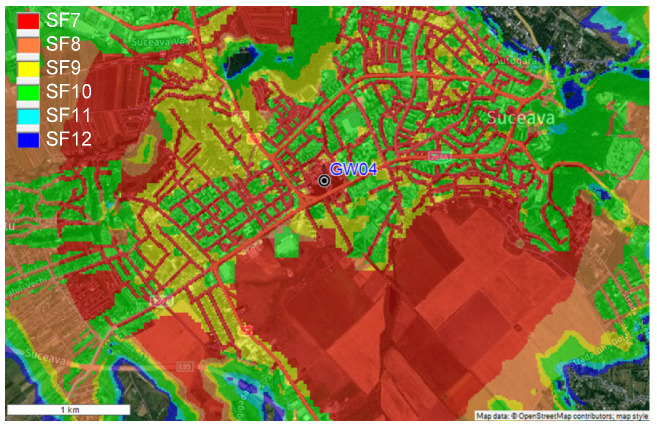
Field coverage measurements—single GW configuration.

From the obtained data depicted in Figure 7, the optimal placement of the GW module for the field measurement tests is on the roof top of Suceava County Hospital (GW04 from the Figure 4), at a height of 360 m above sea level. In this configuration, a single GW will cover about 5 km^2^ in the urban environment, so with a small number of GWs the surface of an entire city may be covered. RadioPlanner can be used by LoRaWAN integrators to determine the optimal placement of the GW modules, thus will reduce the implementation costs and the performance level of the wireless sensor networks.

**Figure 8:**
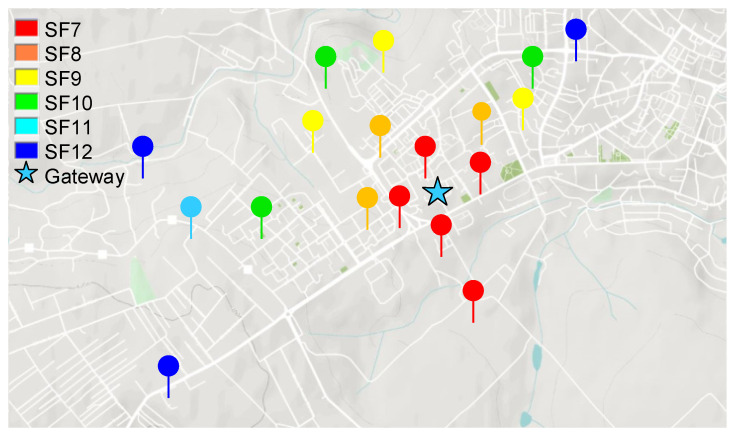
Field coverage measurements of the LoRaWAN gateways installed on the roof top of Suceava Hospital.

The next step was to perform field measurements as to validate the simulated coverage measurements. In this test setup, a RakWireless 7249 gateway [44] was used with an omnidirectional antenna having gain of 12 dBi. Figure 8 presents the obtained results. One can see that the recorded values are very close to the values obtained from our radio propagation model.

**Figure 9:**
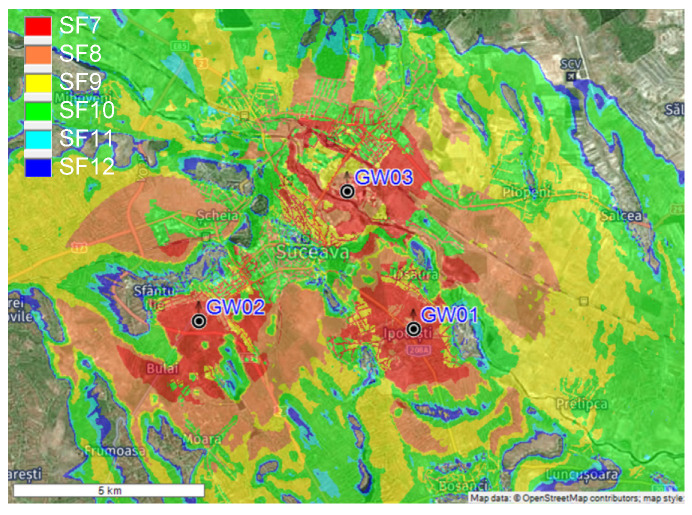
Field coverage measurements—multiple GW configuration.

If the IoT multi-sensor system is to be extended to cover Suceava town entirely, we need to place multiple gateways. Thus, we evaluated the scenario to find out the number of LoRaWAN gateways needed to cover the entire area. As seen in Figure 9, the highest level of performance is obtained when using three gateways: GW01, GW02 and GW03. The distance between the gateways is approximately 5 km and can ensure a coverage area of approximately 20 km^2^, including the neighboring villages.

**Figure 10:**
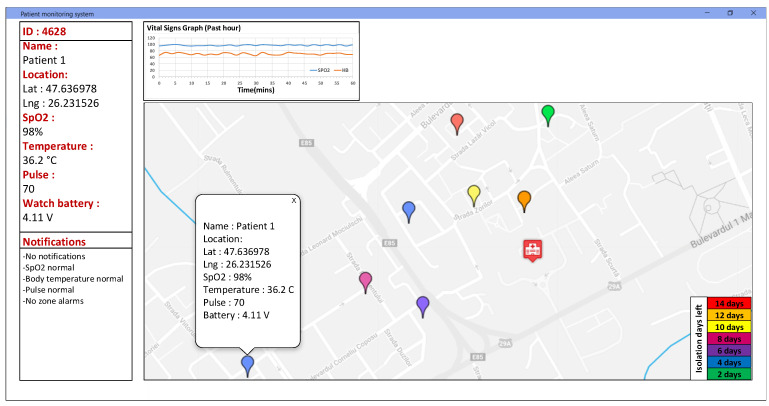
IoT multi-sensor patient monitoring application dashboard.

The GW used in the field tests of the system runs ChirpStack Gateway OS [45], which has the capabilities to decode, store and forward the LoRaWAN packets to external applications under JSON application format. Figure 10 presents the monitoring and control application user interface of the application dashboard. The application is developed as an interactive map that can be easily displayed to the medical staff. The information shown includes the GPS location of each individual patient, his/her vital signals in a build-in graph window, the battery level of the device, the isolation period for each patient and specific alarms based on the threshold setup by the medical staff in the configuration settings of the integrated system.

**Figure 11:**
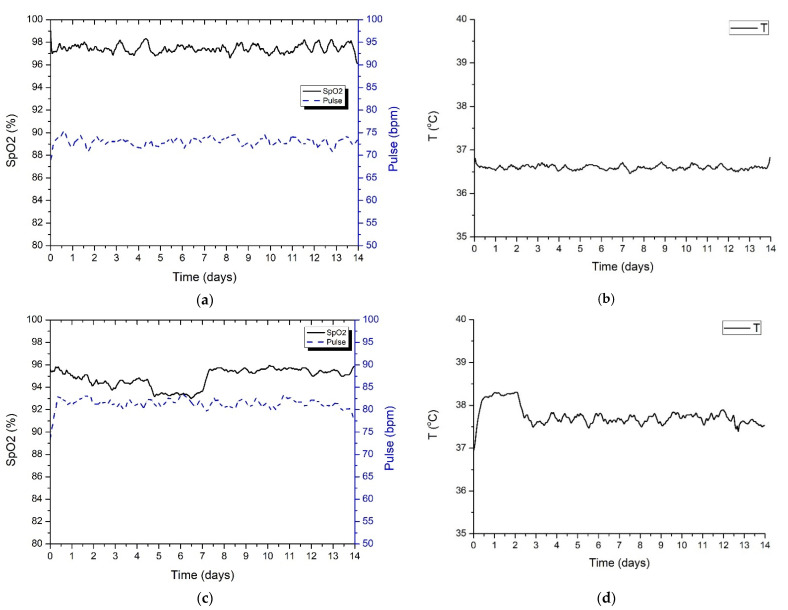
Patients’ vital signs measurement obtained from the IoT wearable device. (**a**) SpO_2_ and pulse (Patient A). (**b**) Body temperature (Patient A). (**c**) SpO_2_ and pulse (Patient B). (**d**) Body temperature (Patient B).

Figure 11 presents the information acquired by the wearable IoT device for two different patients under quarantine. The measurements were acquired for the whole 14-day standard quarantine time. For the first patient, Patient A (Figure 11a,b), the measurements were within normal defined boundaries, while the second patient, Patient B (Figure 11c,d), showed an increase in body temperature and a slight drop in SpO_2_ level, which is associated with a mild form of COVID-19 illness. Further tests and examinations performed on Patient B showed that he was positive for SARS-CoV2 virus infection.

## 5. Conclusions

COVID-19 is caused by the infection of the SARS-CoV2 virus and can be a potentially fatal disease that currently threatens humanity as a global public health issue. The rapid person-to-person transmission of the infection is extremely concerning; thus, a collective effort is needed to find new innovative solutions that can save lives and stop the spread of the disease. Nowadays, the IoT ecosystem has become an extension of the Internet, its impact on how humanity approaches the current problems will be huge in the years to come. This paper presents the design, development, and implementation of an integrated system for COVID-19 illness management. The increase in the number of COVID-19 infected people brought along with it a general overload of the medical system. In many world regions, the hospitals are overwhelmed with severe infection cases, leaving no room for the mild cases or other common diseases.

The paper also analyzes the main IoT challenges regarding the SARS-CoV2 pandemic and presents an in-depth performance evaluation of the IoT communication protocols that can be used for pandemic control. The designed IoT wearable device can monitor the health condition-related parameters like pulse, blood oxygen saturation and body temperature, as well as the patient’s current location. The system can offer great advantages, allowing for remote monitoring of COVID-19 symptoms. 

For testing the proposed system performance, two persons under quarantine were monitored, for a complete 14-day standard quarantine time interval. Based on the data transmitted to the monitoring center, the medical staff decided, after several days of monitoring, when the measured values were out of the normal range, to do an RT-PCR test for one of the two persons, confirming the SARS-CoV2 virus infection. The field tests evaluated scenarios also took into account the optimum placement of the LoRaWAN gateway to obtain the highest level of performance.

The proposed solution that uses the LoRaWAN communication protocol is scalable and can oversee a large number of patients. Table 3 contains a summary comparison of our proposed IoT multi-sensor approach and other systems presented in the scientific literature [27,28,29,30]. The wearable device uses an IoT multi-sensor approach that is easily reconfigurable, offering a flexible and cost-efficient solution for local authorities in the fight against COVID-19 pandemic. The data collected using the developed IoT wearable device can help us to fully understand the spread of the SARS-CoV2 virus. 

## Figures and Tables

**Table 1:** IoT communication protocols that can be implemented in patient monitoring system.

Application Requirements	SigFox	NB-IoT	Symphony Link	IEEE 802.15.4	Z-Wave	IEEE 802.15.1	Cellular Technologies	LoRaWAN
Communication range	~4 km	~1 km	~5 km	~150 m	~100 m	~100 m	~1 km	~5–10 km
Large-scale scalability	✓	✓	✓	-	-	-	✓	✓
Popularity	✓	✓	-	-	✓	✓	✓	✓
Frequency band	Unlicensed	Licensed	Unlicensed	Unlicensed	Unlicensed	Unlicensed	Licensed	Unlicensed
Carrier independent	-	-	✓	✓	✓	✓	-	✓
Power consumption Efficiency	Low	Medium	Low	Low	Low	Low	High	Low
Fast deployment	-	-	-	✓	✓	✓	-	✓
Cost-effective solution	-	-	-	-	-	✓	-	✓
Resistance to interferences	✓	-	-	-	-	✓	✓	✓

**Table 2:** COVID-19 symptom classes.

Patient Class	SpO_2_	Cough Rate	Heartbeat	Temperature
Non-symptomatic	≥95%	No cough	≤90 bpm	≤37.2 °C
Mild symptoms	≥95%	≤5/min	≤100 bpm	36 °C ≤ *T* ≤ 38 °C
Moderate clinical symptoms	93% ≤ SpO_2_ ≤ 94%	5/min ≤ Cough Rate < 30/min	>100 bpm	≥38 °C
Serious clinical symptoms	≤92%	≥30/min	>120 bpm	>38 °C

**Table 3:** Performance evaluation of the developed system compared with other solutions.

Application Parameters	Zhang et al. [27]	Ullah et al. [28]	Mukhtar et al. [29]	Hoang et al. [30]	Our Proposed Approach
SpO_2_	-	✓	✓	-	✓
Pulse	-	✓	✓	-	✓
Temperature	✓	✓	✓	✓	✓
Location	-	✓	-	-	✓
Scalability of the system	-	-	-	-	✓
High coverage area	-	-	-	-	✓
Modular architecture	-	-	-	-	✓
Wearable device integration	-	-	-	✓	✓
IoT communication technology	BLE	BLE/802.11n	802.11	BLE/802.11n	LoRaWAN
